# Supplementary material for: A single-cell multiomics roadmap of zebrafish spermatogenesis reveals regulatory principles of male germline formation
Source: Mol Syst Biol. 2025 Oct 14;22(1):42–68. doi: 10.1038/s44320-025-00157-7 (PMC12759076; doi:10.1038/s44320-025-00157-7)
Supplement: Supplementary file 1 — Appendix [file 44320_2025_157_MOESM1_ESM.pdf]

# Appendix for:

# A single-cell multiomics roadmap of zebrafish spermatogenesis reveals regulatory principles of male germline formation

Ana María Burgos Ruíz[1], Fan-Suo Geng[2], Gala Pujol[3,4], Estefanía Sanabria[1], Thirsa Brethouwer[1], María Almuedo-Castillo[1], Aurora Ruiz-Herrera[3,4] Juan J. Tena[1,*], Ozren Bogdanovic[1,2,5,*]

1 Centro Andaluz de Biología del Desarrollo, CSIC-Universidad Pablo de Olavide-Junta de Andalucía, Seville, Spain
2 Garvan Institute of Medical Research, Sydney, New South Wales, Australia
3 Institut de Biotecnologia i Biomedicina, Universitat Autònoma de Barcelona, Cerdanyola del Vallès, Spain
4 Departament de Biologia Cel·lular, Fisiologia i Immunologia, Universitat Autònoma de Barcelona, Cerdanyola del Vallès, Spain
5 School of Biotechnology and Biomolecular Sciences, University of New South Wales, Sydney, NSW, Australia
* Correspondence to o.bogdanovic@csic.es or juan.tena@csic.es

## Table of Contents

Appendix Figure S1........ 1
Appendix Figure S2........ 2
Appendix Figure S3........ 3
Appendix Figure S4........ 4
Appendix Figure S5........ 5
Appendix Figure S6........ 6
Appendix Figure S7........ 7
Appendix Table S1........ 8
Appendix Table S2........ 9

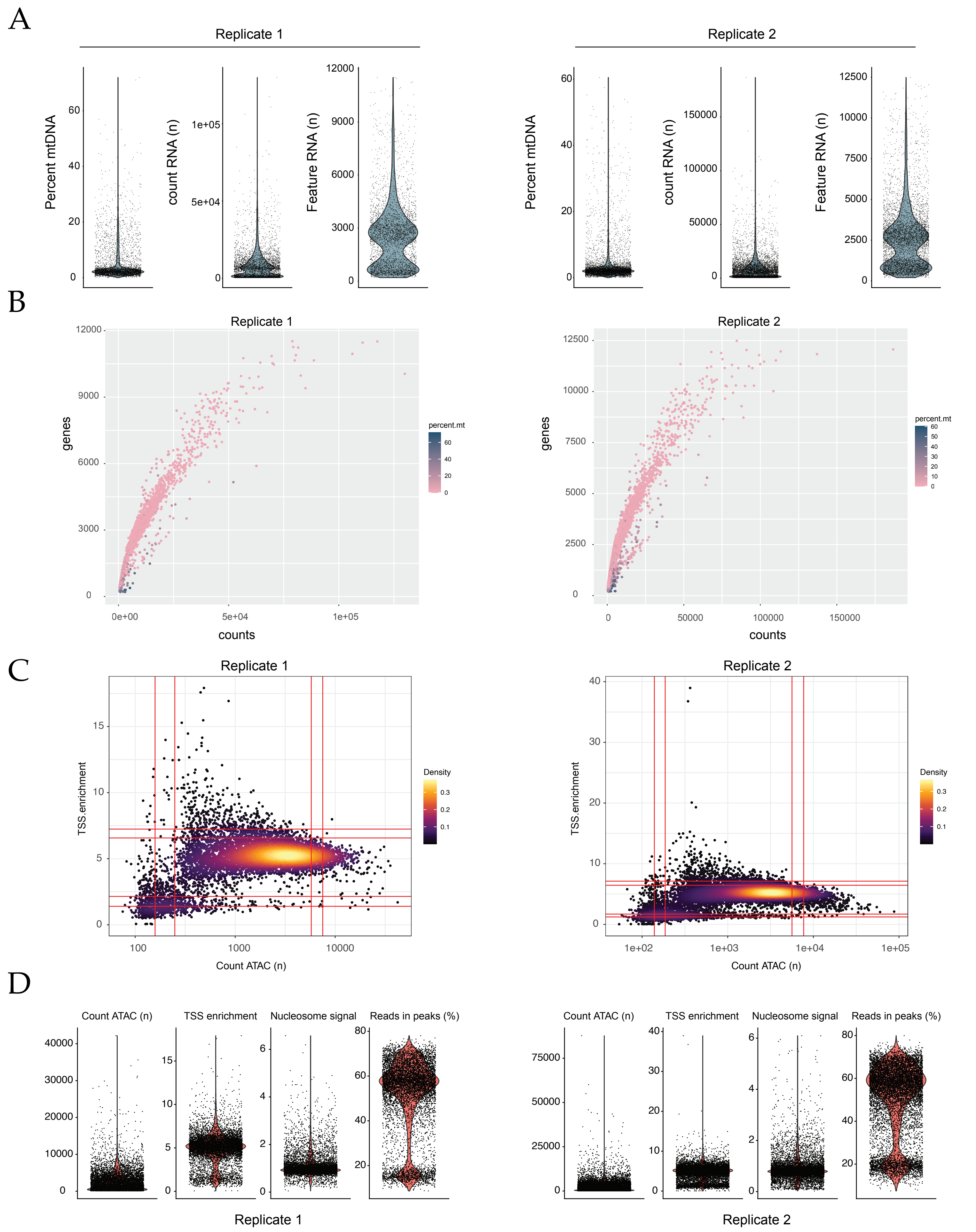


**Appendix Figure S1. scRNA-seq and scATAC-seq quality control. (A)** Distribution of quality control (QC) metrics per cell across replicates, including percentage of mitochondrial reads, total RNA counts per cell, and number of detected features per cell. **(B)** Scatter plot of total RNA counts versus number of detected genes, with color gradient indicating the percentage of mitochondrial reads. **(C)** Density scatter plot showing the relationship between total peak counts per cell and transcription start site (TSS) enrichment in both samples; the x-axis is log-transformed, cell density is indicated by color intensity, and quantile lines are shown to aid in identifying potential QC thresholds. **(D)** Violin plots displaying the distribution of key ATAC-seq QC metrics per cell across replicates, including total peak counts, TSS enrichment, nucleosome signal, and percentage of reads in peaks.

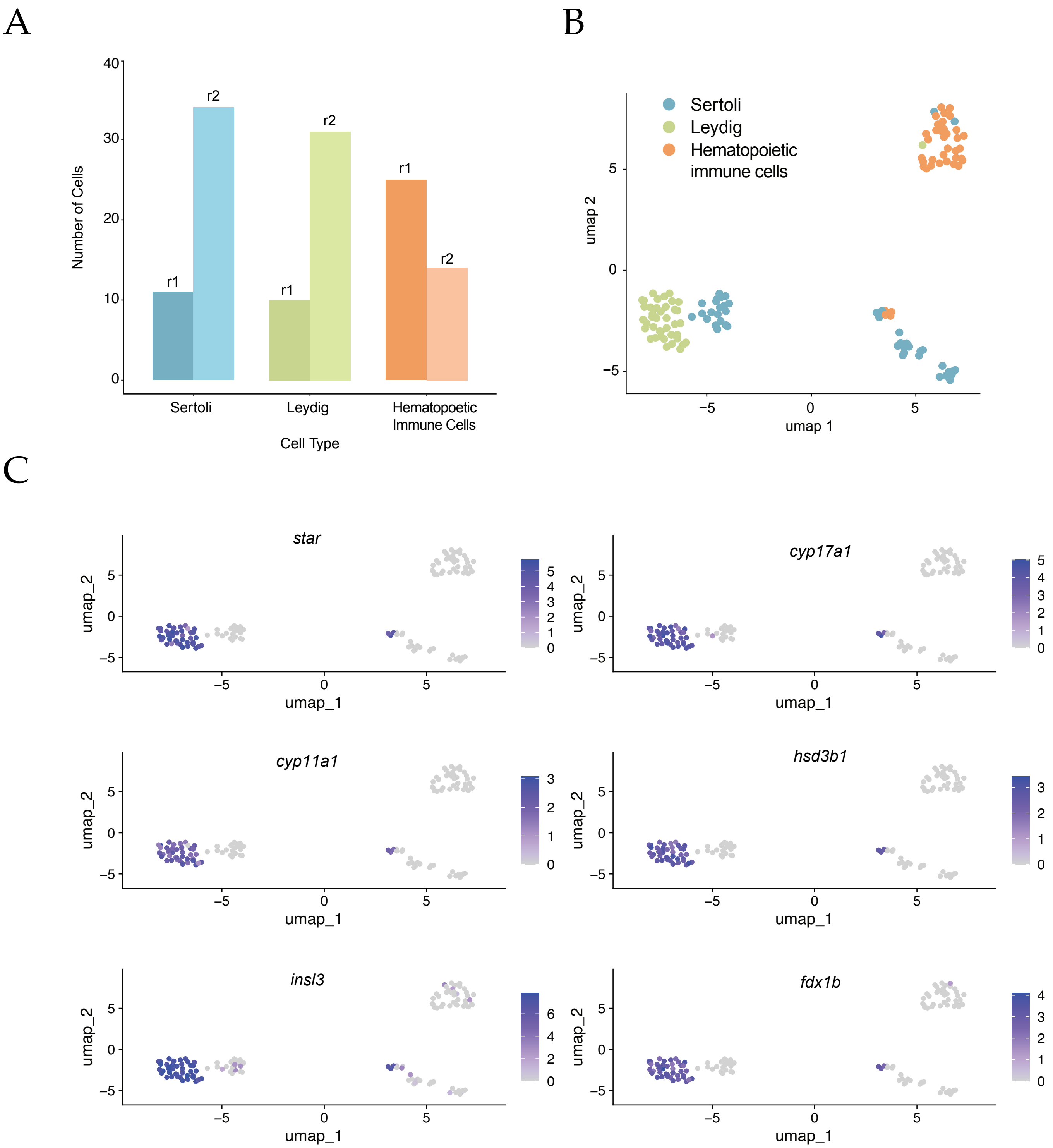


**Appendix Figure S2. Frequency and content of somatic cell poulations found in the zebrafish testis. (A)** Number of identified somatic cells per replicate and cell type. **(B)** UMAP plot showing diverse somatic cell populations: Sertoli, Leyding anf Hematopoetic immune cells. Sertoli cells exist in two major configurations likely depending on activation or spatial location within the testis. **(C)** Expression profiles of Leydig cell marker genes.

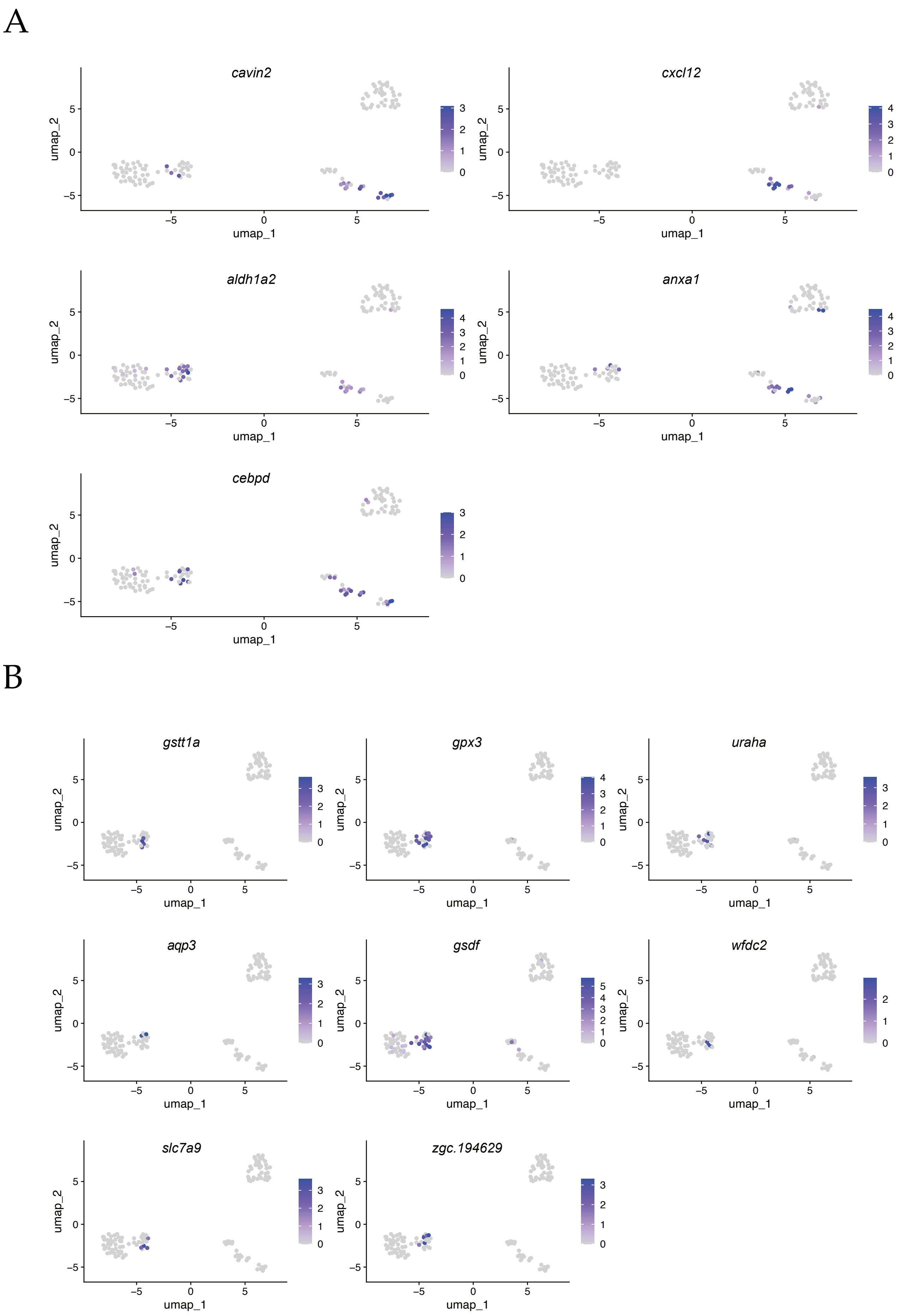


**Appendix Figure S3. UMAP expression plots of Sertoli cell markers. (A)** Expression profiles of canonical Sertoli marker genes including *aldh1a2*, *cxcl12*, *anxa1* and others. **(B)** Expression profiles of marker genes associated with a specialised Sertoli cell population possibly reflecting activation state or spatial positioning within the testis.

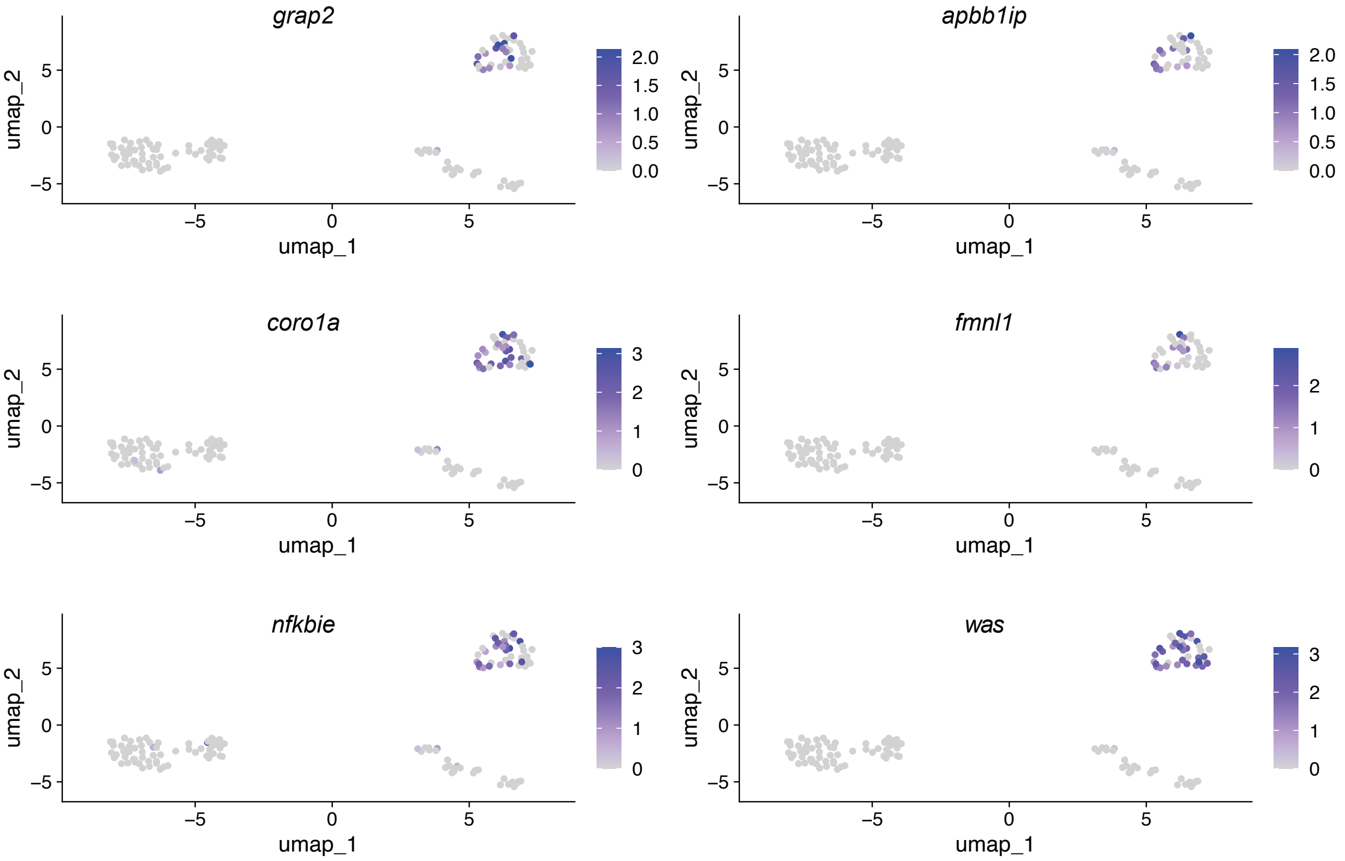


**Appendix Figure S4. UMAP expression plots of hematopoetic immune cell markers.** Expression profiles of canonical hematopoetic immune cell markers such as *grap2*, *coro1a* and others.

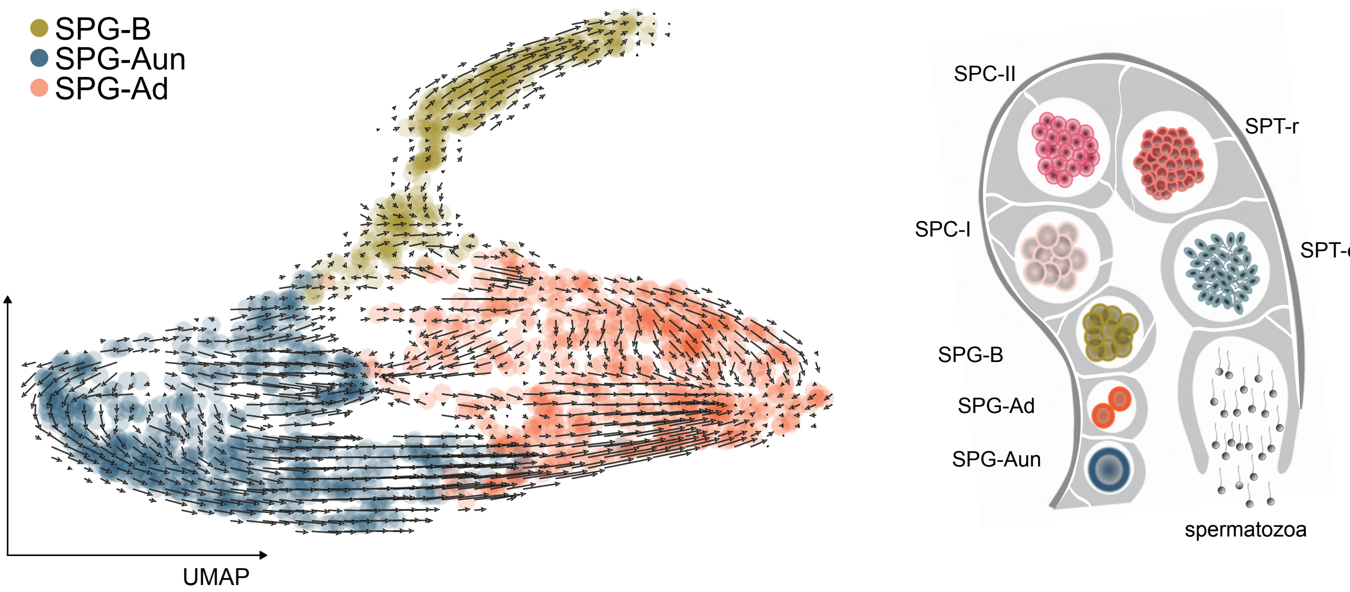


**Appendix Figure S5. SPG Differentiation trajectories.** RNA velocity analysis based on the relative abundance of unspliced and spliced mRNA in scRNA-seq SPG populations. Velocity vectors, representing the predicted direction and magnitude of transcriptional progression, are overlaid on the UMAP, revealing distinct transcriptional trajectories within the spermatogonial compartment. Samples: undifferentiated spermatogonia-A (SPG-Aun), differentiated spermatogonia-A (SPG-Ad), spermatogonia B (SPG-B). Right panel - schematic drawing of the spermatogenesis process in *Danio rerio*.

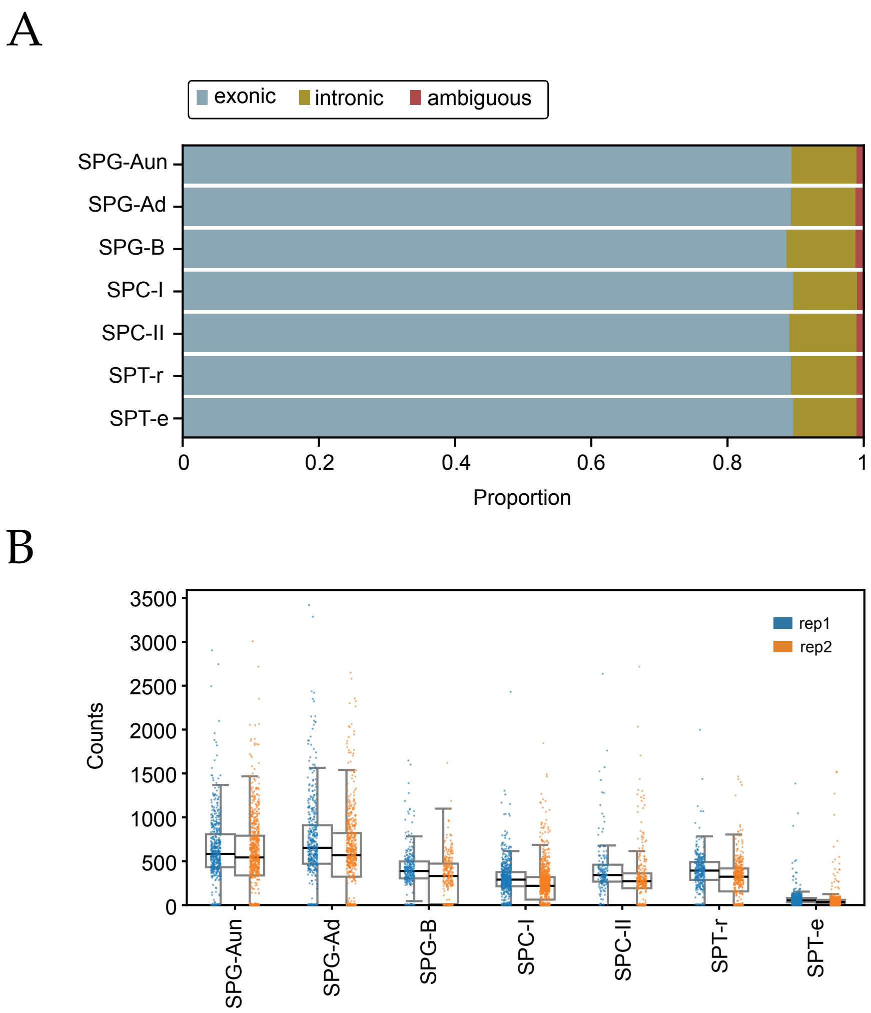


**Appendix Figure S6. RNA turnover dynamics during zebrafish spermatogenesis. (A)** Velocyto-derived proportions of exonic, intronic, and ambiguous reads across germ cell populations. All populations, from undifferentiated spermatogonia (SPG-Aun) to elongated spermatids (SPT-e). **(B)** Total intronic read counts per cell across biological replicates (rep1: blue; rep2: orange). Boxplots represent medians and interquartile ranges; individual data points correspond to single cells.

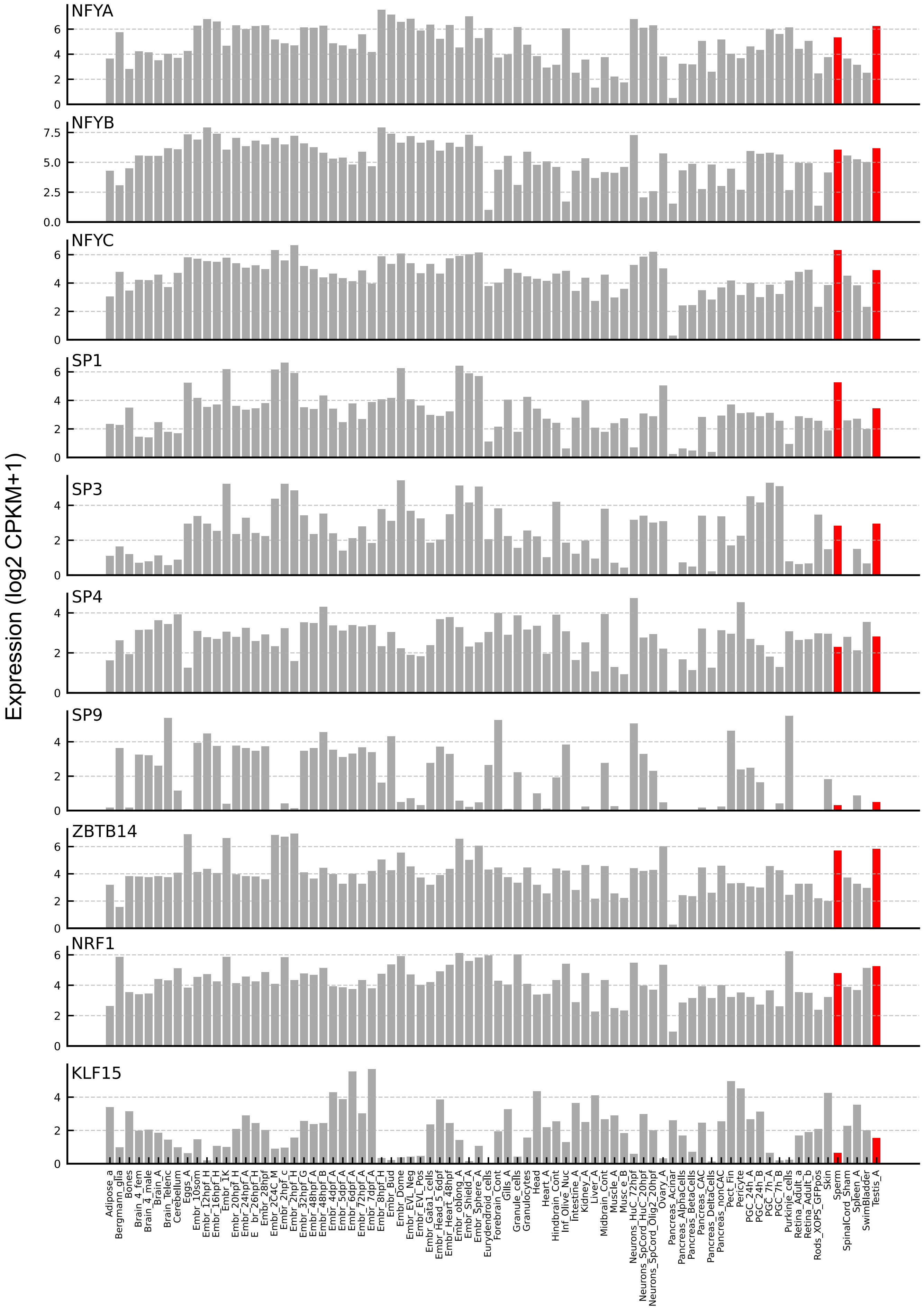


**Appendix Figure S7.** Expression profiles of CGI-associated chromatin proteins. Expression levels are shown as $\log_2$(CPKM+1), where CPKM refers to counts per kilobase of transcript per million mapped reads. Data are taken from embryonic, somatic, and germline tissues as compiled in the VastDB database (https://vastdb.crg.eu/). Testis and sperm samples are marked in red for clarity.

| Annotation | log2 ratio obs/exp | (-)log *p* val |
|---|---|---|
| CpG-Island | 3018 | 77352 |
| tRNA | 1768 | 2819 |
| LTR | 1013 | 79.29 |
| 5UTR | 585 | 1279 |
| Promoter | 55 | 885 |
| Exon | -66 | -928 |
| snRNA | -88 | -63 |
| pseudo | -0.09 | -65 |
| rRNA | -121 | -88 |
| ncRNA | -212 | -158 |
| TTS | -303 | -2206 |
| Satellite | -388 | -3543 |
| Simple repeat | -456 | -7814 |
| Intergenic | -631 | -122.49 |
| Low complexity | -663 | -2159 |
| LINE | -708 | -10814 |
| 3UTR | -1175 | -5501 |
| Intron | -1396 | -144798 |
| Satellite? | -1.51 | -1847 |
| SINE | -1912 | -44.84 |

**Appendix Table S1. Genomic feature enrichment of differentially methylated regions (DMRs).** Enrichment analysis of DMRs across various annotated genomic features. Ratio ($\log_2$) of observed versus expected overlaps between DMRs and each genomic category (positive values indicate enrichment; negative values indicate depletion), along with the $-\log_{10}$ transformed *p* value from the hypergeometric test. Enrichment was computed using HOMER, comparing the distribution of DMRs to the genomic background.

| Sample 1: Filter | Cells retained | Cells discarded |
|---|---|---|
| initial | 7411 | 0 |
| nCount_ATAC > 500 | 5783 | 1628 |
| nCount_ATAC < 10000 | 5619 | 164 |
| pct_reads_in_peaks > 40 | 5273 | 346 |
| nucleosome_signal < 1 | 3637 | 1636 |
| TSS.enrichment > 5 | 2372 | 1265 |
| scDblFinder_doublets == "singlet" | **2099** | 273 |
| **Sample 2: Filter** | **Cells retained** | **Cells discarded** |
| initial | 9898 | 0 |
| nCount_ATAC > 500 | 7457 | 2441 |
| nCount_ATAC < 10000 | 7171 | 286 |
| pct_reads_in_peaks > 40 | 6556 | 615 |
| nucleosome_signal < 1 | 6038 | 518 |
| TSS.enrichment > 5 | 3964 | 2233 |
| scDblFinder_doublets == "singlet" | **3251** | 554 |

**Appendix Table S2. Quality control and filtering of scATAC-seq datasets.** Columns indicate the number of cells retained and discarded at each step. Initial barcodes were first filtered by total fragment count (nCount_ATAC > 500 and < 10,000), followed by removal of cells with low signal-to-noise ratios based on the percentage of reads in peaks (pct_reads_in_peaks > 40%), nucleosome banding pattern (nucleosome_signal < 1), and transcription start site (TSS) enrichment (TSS.enrichment > 5). Doublets were identified and removed using scDblFinder, retaining only barcodes classified as singlets. These thresholds were chosen based on standard guidelines for high-confidence scATAC-seq preprocessing.
