# Supplementary material for: A single-cell multiomics roadmap of zebrafish spermatogenesis reveals regulatory principles of male germline formation
Source: Mol Syst Biol. 2025 Oct 14;22(1):42–68. doi: 10.1038/s44320-025-00157-7 (PMC12759076; doi:10.1038/s44320-025-00157-7)
Supplement: Supplementary file 18 — Expanded View Figures [file 44320_2025_157_MOESM18_ESM.pdf]

Expanded View Figures

Figure EV1. scRNA-seq quality control, marker genes and GO enrichments.

(**A**) Quality control metrics for scRNA-seq replicates showing the relationship between counts, detected gene numbers, and mitochondrial DNA percentage. (**B**) UMAP (uniform manifold approximation and projection) plots of the zebrafish testis tissue denoted by Seurat clusters before filtering. (**C**) Distribution of average counts, and average gene numbers across the spermatogenic lineage. (**D**) Expression UMAP for canonical marker genes for SPG (*ddx4*), SPG-Aun (*eno3*, *e2f5* and *ripply2*), SPG-Ad(*hist1h2a6*), SPT-e (*edrf1*), SPC (*sycp3*) and proliferation (*pcna*). (**E**) Dot plot of the top 5 GO biological processes enriched using marker genes for each cell type. The x-axis shows adjusted *P* values (FDR), and dot size reflects the ratio of intersected to total terms. Enrichment *P* values were computed with Fisher's exact one-tailed test (cumulative hypergeometric).

 © The Author(s)

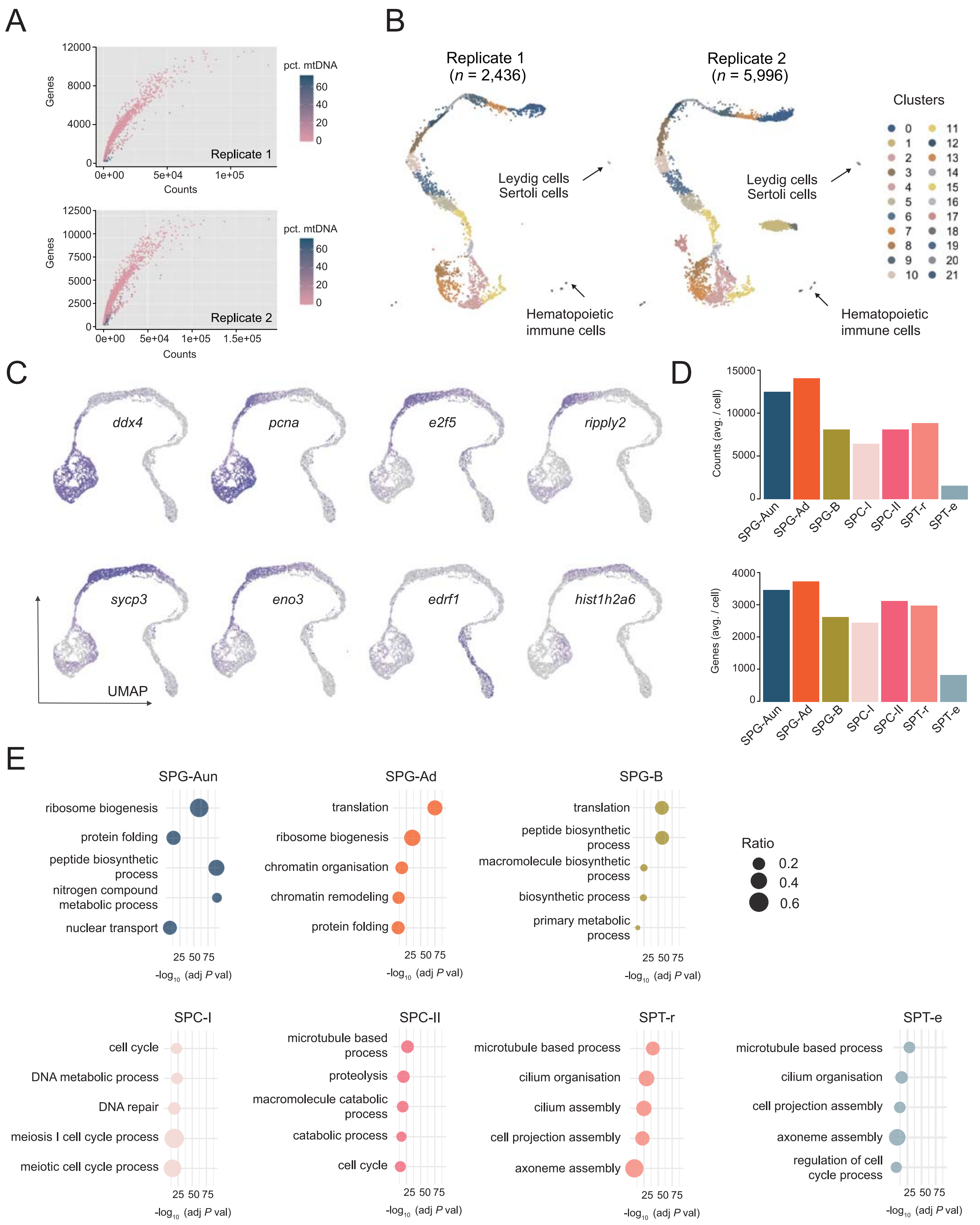
A
Genes
Counts
pct. mtDNA
Replicate 1
Replicate 2
B
Replicate 1
(n = 2,436)
Replicate 2
(n = 5,996)
Clusters
Leydig cells
Sertoli cells
Hematopoietic
immune cells
C
ddx4
pcna
e2f5
ripply2
sycp3
eno3
edrf1
hist1h2a6
UMAP
D
Counts (avg. / cell)
Genes (avg. / cell)
SPG-Aun
SPG-Ad
SPG-B
SPC-I
SPC-II
SPT-r
SPT-e
E
SPG-Aun
ribosome biogenesis
protein folding
peptide biosynthetic process
nitrogen compound metabolic process
nuclear transport
SPG-Ad
translation
ribosome biogenesis
chromatin organisation
chromatin remodeling
protein folding
SPG-B
translation
peptide biosynthetic process
macromolecule biosynthetic process
biosynthetic process
primary metabolic process
Ratio
0.2
0.4
0.6
SPC-I
cell cycle
DNA metabolic process
DNA repair
meiosis I cell cycle process
meiotic cell cycle process
SPC-II
microtubule based process
proteolysis
macromolecule catabolic process
catabolic process
cell cycle
SPT-r
microtubule based process
cilium organisation
cilium assembly
cell projection assembly
axoneme assembly
SPT-e
microtubule based process
cilium organisation
cell projection assembly
axoneme assembly
regulation of cell cycle process
-log10 (adj P val)

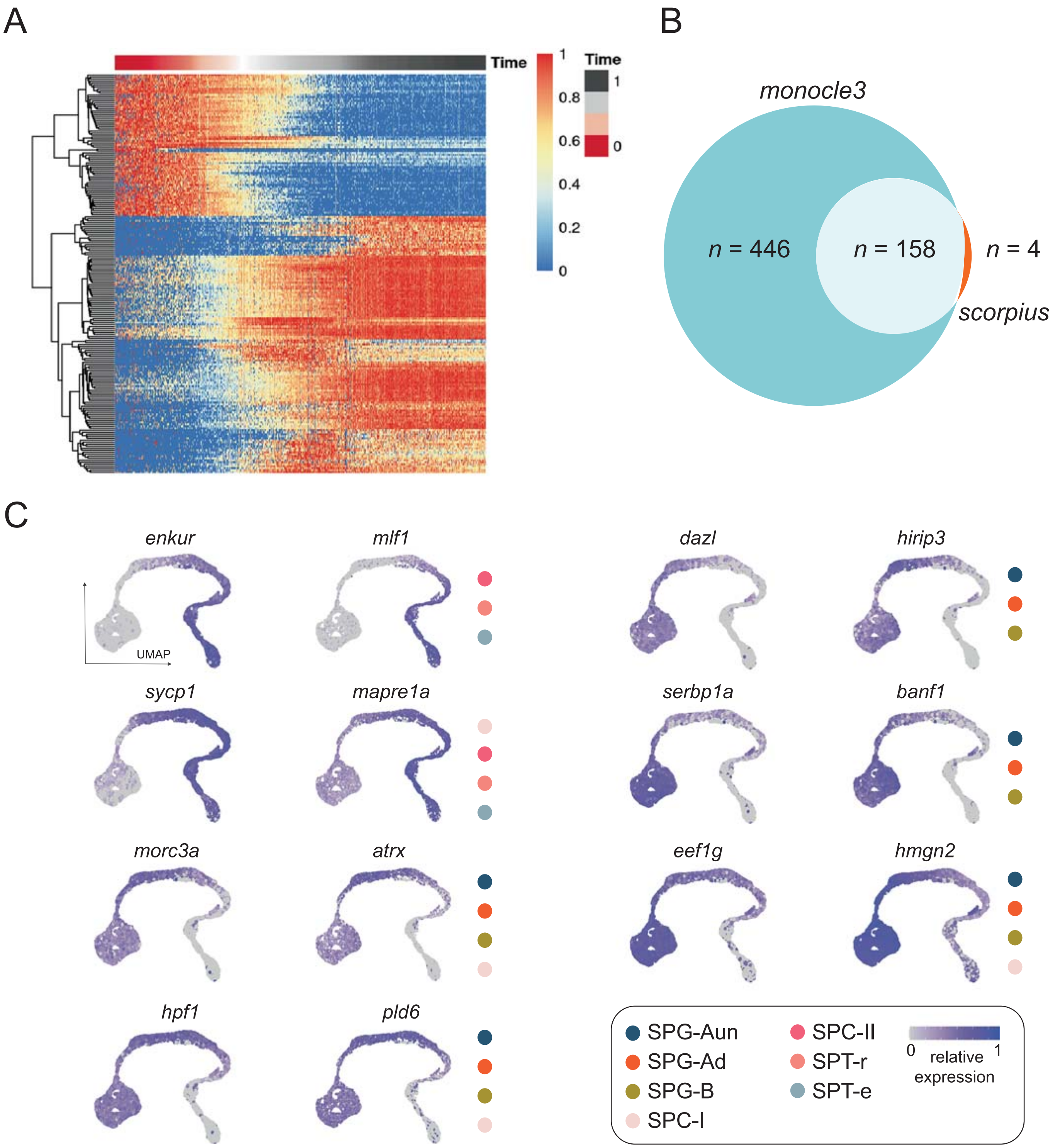

Figure EV2. Trajectory analysis and expression profiles of spermatogenesis driver genes.

(**A**) Heatmap of drive genes across the inferred trajectory of spermatogenesis. Samples ranked by pseudotime trajectory inferred by SCORPIUS. Rows represent genes grouped by hierarchical clustering with distinct expression patterns. (**B**) Comparison of gene drivers for spermatogenesis calculated using Monocle3 and SCORPIUS software. (**C**) UMAP examples for gene drivers obtained from the trajectory analysis identified by both Monocle3 and SCORPIUS.

© The Author(s)

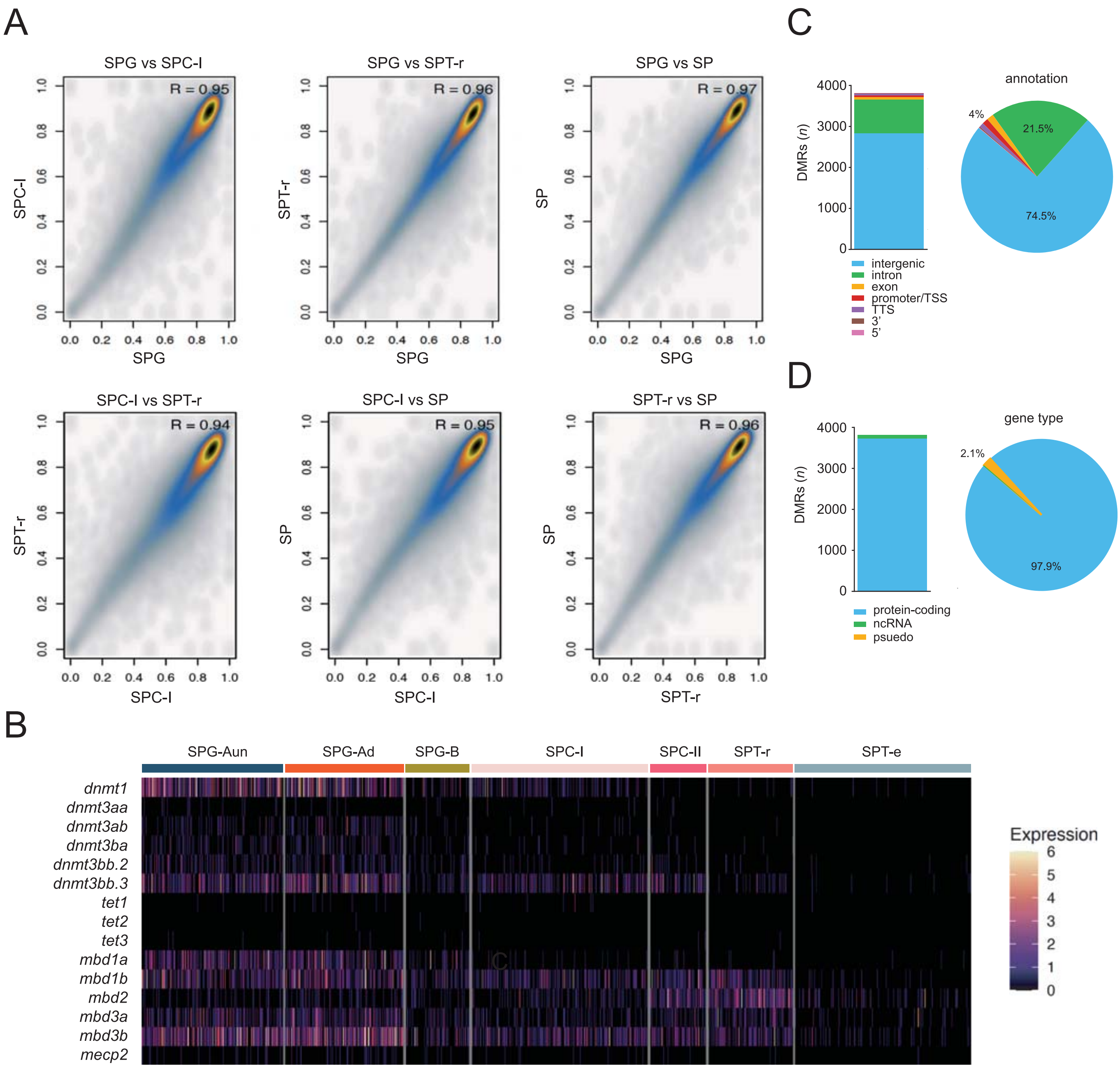

Figure EV3. DNA methylomes and genomic localisation of Differentially Methylated Regions (DMRs).

(**A**) Correlation of CpG methylation levels in non-overlapping 10 Kb genomic bins in isolated germ cell populations (SPG: spermatogonia, SPC-I: spermatocytes I, SPT-r: round spermatids, and mature sperm: SP). *R* value represents Pearson Correlation coefficient. SPG vs SPC-I, SPC-I vs SPT-r, and SPT-r vs SP comparisons are also shown in Fig. 3E. (**B**) Single-cell RNA-seq expression of DNA methylation, deposition removal, and interpretation machinery during zebrafish spermatogenesis. Genes include DNA methyltransferases, TET dioxygenases, and methyl-CpG-binding domain proteins. Expression is shown across major germ cell populations, ordered by developmental progression from undifferentiated spermatogonia to mature spermatids. (**C**) Genomic localisation of DMRs ($n = 3879$) represented as absolute values (left panel) and percentages (pie chart, right panel) identified between four germ cell populations. (**D**) Gene type enrichment of the identified DMRs.

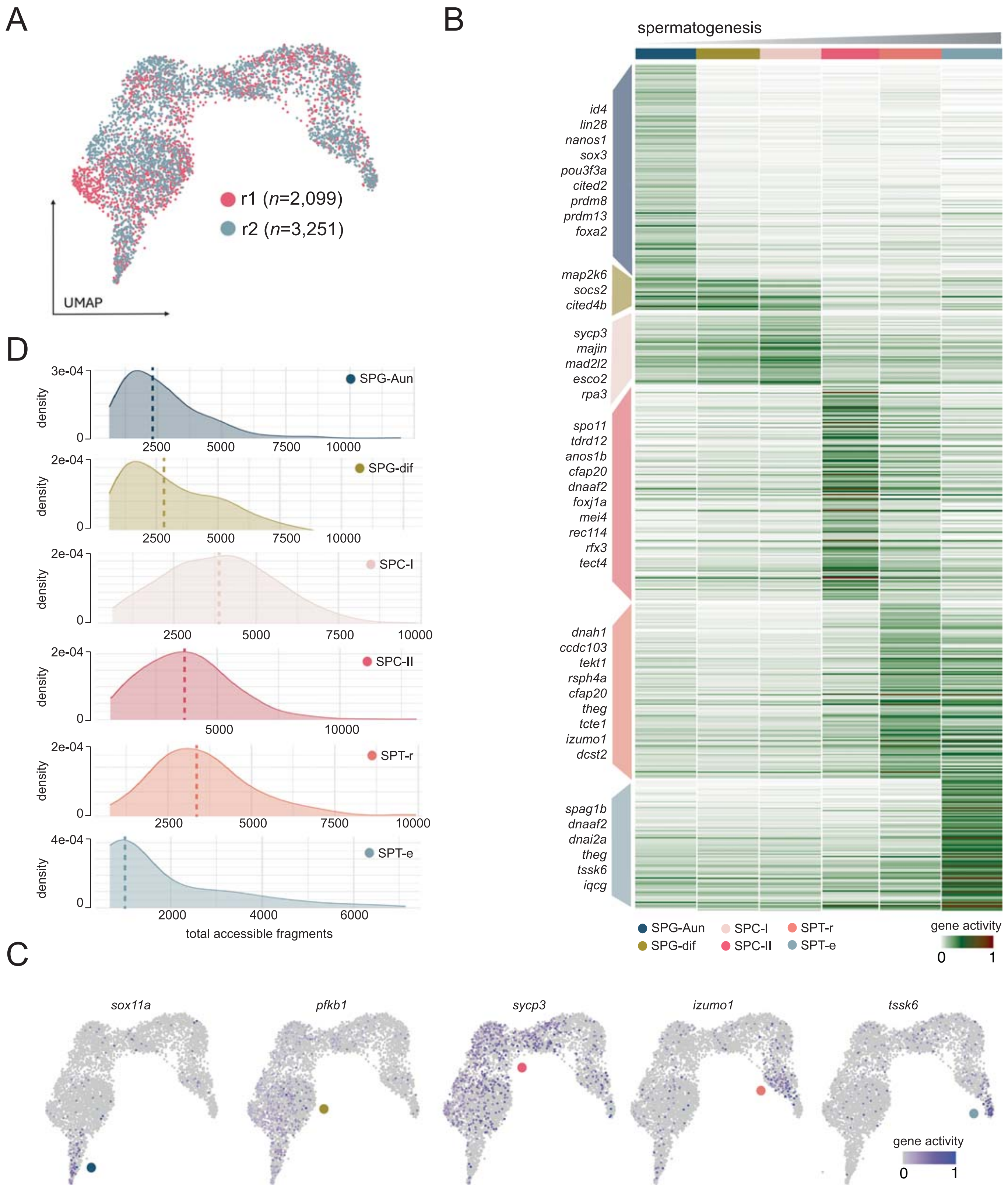
A
r1 (n=2,099)
r2 (n=3,251)
UMAP
B
spermatogenesis
id4
lin28
nanos1
sox3
pou3f3a
cited2
prdm8
prdm13
foxa2
map2k6
socs2
cited4b
sycp3
majin
mad2l2
esco2
rpa3
spo11
tdrd12
anos1b
cfap20
dnaaf2
foxj1a
mei4
rec114
rfx3
tect4
dnah1
ccdc103
tekt1
rsph4a
cfap20
theg
tcte1
izumo1
dcst2
spag1b
dnaaf2
dnai2a
theg
tssk6
iqcg
SPG-Aun
SPG-dif
SPC-I
SPC-II
SPT-r
SPT-e
gene activity
0
1
D
density
3e-04
2e-04
4e-04
0
2500
5000
7500
10000
2000
4000
6000
total accessible fragments
C
sox11a
pfkb1
sycp3
izumo1
tssk6
gene activity
0
1

 © The Author(s)

Figure EV4. Single cell chromatin accessibility (scATAC-seq) dynamics during zebrafish spermatogenesis.

(**A**) UMAP (uniform manifold approximation and projection) of biological replicates of scATAC-seq data. Cells are coloured by replicate label. (**B**) Sorted heatmap of averaged gene expression profiles grouped by cell type (SPG-Aun, SPG-dif, SPC-I, SPC-II, SPT-r, SPT-e). Top marker genes for each cluster are indicated on the left-hand side of the heatmap. (**C**) UMAP plots showing gene activity of marker gene examples associated with spermatogenesis: *sox11* - undifferentiated spermatogonia; *pfkb1* - differentiated spermatogonia; *sycp3* - spermatocytes; *izumo1* - round spermatids; *tssk6* - elongated spermatids. Gene activity is defined as the normalised sum of fragments spanning the gene body and extending 2 kb upstream of the transcription start site. (**D**) Distribution of the number of accessible genome fragments in each cell type (SPG-Aun, SPG-dif, SPC-I, SPC-II, SPT-r, SPT-e). The median is indicated by the dashed line.

© The Author(s)

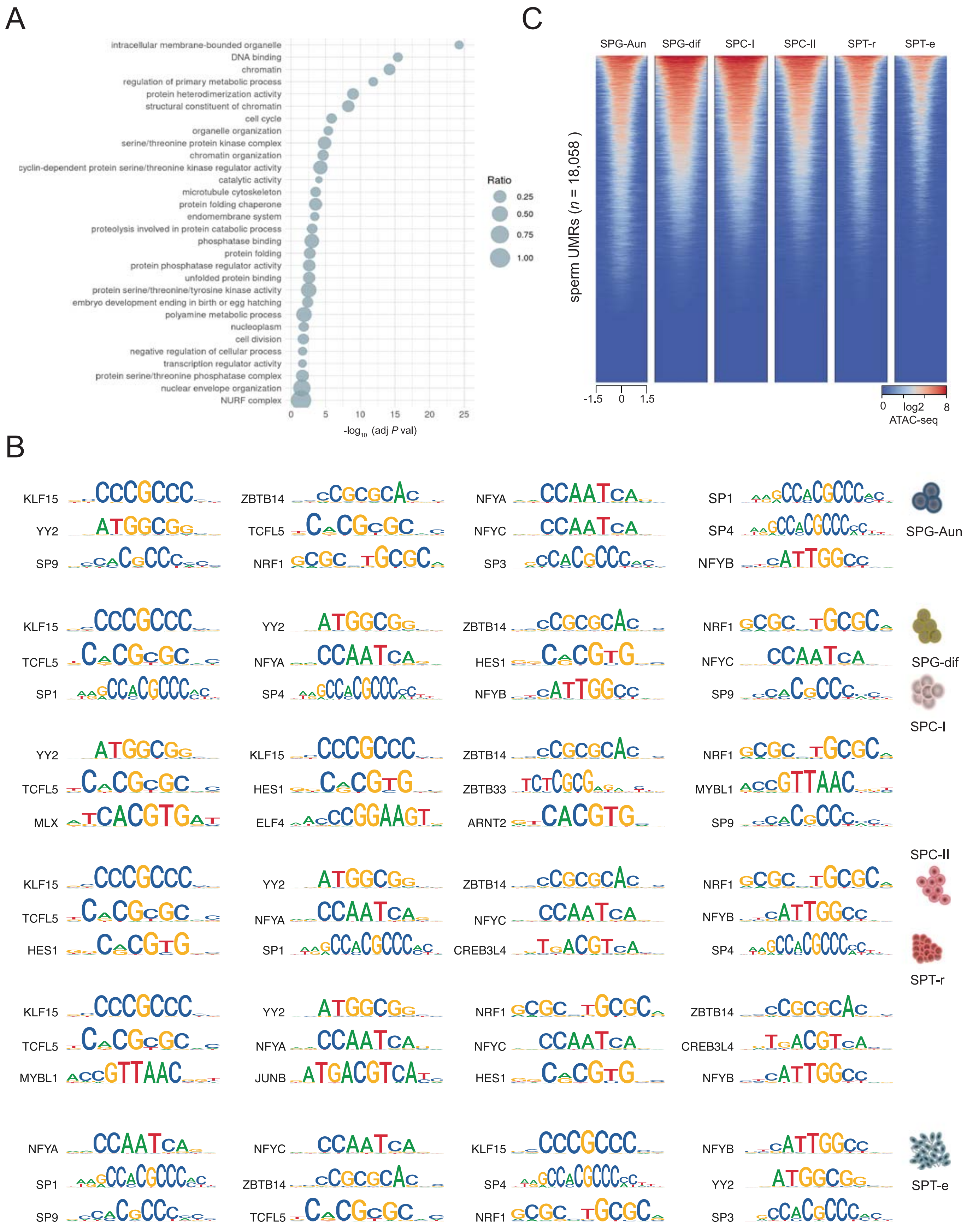
A
intracellular membrane-bounded organelle
DNA binding
chromatin
regulation of primary metabolic process
protein heterodimerization activity
structural constituent of chromatin
cell cycle
organelle organization
serine/threonine protein kinase complex
chromatin organization
cyclin-dependent protein serine/threonine kinase regulator activity
catalytic activity
microtubule cytoskeleton
protein folding chaperone
endomembrane system
proteolysis involved in protein catabolic process
phosphatase binding
protein folding
protein phosphatase regulator activity
unfolded protein binding
protein serine/threonine/tyrosine kinase activity
embryo development ending in birth or egg hatching
polyamine metabolic process
nucleoplasm
cell division
negative regulation of cellular process
transcription regulator activity
protein serine/threonine phosphatase complex
nuclear envelope organization
NURF complex
0 5 10 15 20 25
$-\log_{10}$ (adj P val)
Ratio
0.25
0.50
0.75
1.00
C
SPG-Aun SPG-dif SPC-I SPC-II SPT-r SPT-e
sperm UMRs (n = 18,058)
-1.5 0 1.5
0 log2 8
ATAC-seq
B
KLF15 ZBTB14 NFYA SP1
YY2 TCFL5 NFYC SP4
SP9 NRF1 SP3 NFYB
SPG-Aun
KLF15 YY2 ZBTB14 NRF1
TCFL5 NFYA HES1 NFYC
SP1 SP4 NFYB SP9
SPG-dif
SPC-I
YY2 KLF15 ZBTB14 NRF1
TCFL5 HES1 ZBTB33 MYBL1
MLX ELF4 ARNT2 SP9
SPC-II
KLF15 YY2 ZBTB14 NRF1
TCFL5 NFYA NFYC NFYB
HES1 SP1 CREB3L4 SP4
SPT-r
KLF15 YY2 NRF1 ZBTB14
TCFL5 NFYA NFYC CREB3L4
MYBL1 JUNB HES1 NFYB
NFYA NFYC KLF15 NFYB
SP1 ZBTB14 SP4 YY2
SP9 TCFL5 NRF1 SP3
SPT-e

Figure EV5. Chromatin state of elongated spermatids (SPT-e).

(**A**) Dot plot of top biological processes associated with genes linked to open chromatin peaks in the SPT-e population. The x-axis shows adjusted *P* values (FDR), and dot size reflects the ratio of intersected to total terms. Enrichment *P* values were computed with Fisher's exact one-tailed test (cumulative hypergeometric). (**B**) Top 12 enriched motifs found in open chromatin peaks in each cell population. (**C**) ATAC-seq signal (log2) from all spermatogenesis cell types, plotted over unmethylated regions (UMRs) identified in mature sperm.
